# The Institutional Worker

**Published:** 1920-10-09

**Authors:** 


### INSTITUTIONAL WORKER

**Being a Special Supplement to "The Hospital"**

### OUR BUREAU OF INFORMATION.

### Rules for Correspondents

1. Every letter must be accompanied by the coupon to be cut from the back cover (inside page) of The Hospital, current issue, and must contain the name and address of the correspondent with pseudonym for publication if desired. Replies by post cannot be given save under exceptional circumstances at the Editor's discretion.

2. Letters from Approved Homes in reply to special needs published in the Bureau should state terms and full particulars, and be sent prepaid under cover to the Editor of the Bureau with name written across coupon for identification.

3. Proprietors of Homes which have not yet been entered on the List of Approved Homes, but have spare accommodation likely to suit special needs, are invited to write for an application form for registration. The fee for registration, which includes two announcements of the Home in the Bureau and other privileges, is 10s.

4. All communications to be addressed to the Editor of The Hospital, 28 Southampton Street, Strand, London, W.C. 2, and marked "Bureau of Information."

### INSTITUTION FACTS AND FIGURES.

### The Hospital Typist.

**By One Who Has Retired.**

The usefulness and place of the typist in the working community are not sufficiently well known. A really good typist, which term, of course, includes the knowledge and experience of a good stenographer, should possess capabilities varied and unique. She should be able to put into grammatical order sentences of much intricacy, to decipher hieroglyphics (her employer's handwriting) which would baffle an archæologist, and, if she is the only correspondent in the establishment, as frequently is the case, she must be prepared to act as an encyclopædia of general information for the office. In addition to all this, the hospital typist should be able to express herself intelligently upon subjects many and varied; she must be acquainted with the technical terms of a multitude of trades, and be able to correspond on topics ranging from laundry machinery to paints and varnishes, and from *x*-ray apparatus to scrubbing-brushes. She must have the courage of an aviator and the patience of a saint. She must be ever available, always at her post; clerks may come and clerks may go, but she goes on for ever; that is to say, the clerks may shut up their desks and go home, but it is frequently the typist's lot meekly to await the advent of the "Chief" on his return from a busy afternoon in the City; or, perhaps, at the end of the day the general superintendent is deep in a long-sought conference with the resident medical officer, and has completely forgotten her existence, and there she must wait until some happy circumstance reminds him of her presence and of the fact that it is time she had some tea. I hope if any guilty general superintendent reads these lines he will repent in sackcloth and ashes, and resolve to treat his typist with a greater measure of consideration for the future.

Still, the life of a typist has its compensation. There is a certain dignity attached to it for one thing—the dignity of brains. It is no slight task to write in shorthand from dictation, and afterwards type the replies to innumerable letters. The rush of business in a large hospital is sometimes very great, particularly at "special appeal" times, when the cheques are flowing in—metaphorically speaking—through the post, through the window, and over the counter, every hour, and have to be acknowledged the same day (I am, needless to say, speaking of pre-war days).

The typist's position is one of trust, because to her, necessarily, must be imparted secrets of the most private and confidential nature, and she is often in possession of most important facts relating to the working of the hospital. The woman typist has proved her worth in this respect up to the hilt, in spite of the oft-expressed conviction of the male mind that a woman cannot keep a secret. Every business man will tell you that a good typist is worth her weight in gold, and that her price is far above rubies.

Given a good education, a thorough practical knowledge of shorthand and typewriting, and industry, a girl is well equipped for the battle of life, and should always be able to secure a decent livelihood. If, like the busy bee, she improves the shining hour by adding to her stock of knowledge, there is every prospect of her obtaining a really good post. If the hospital typist makes it her business to take an interest in, and become acquainted with, the details of the administration of the hospital, she can become not only valuable to her employer but also a really useful member of the hospital staff.

The life of a hospital typist is not by any means the irksome existence some would seem to suppose. Of course, a great deal depends upon the sphere to which her labours are confined. If it is her misfortune to be the only woman clerk in the establishment, and she is compelled to work in the general office amid the rush of business, the ringing of the telephone, the facetiousness of the office-boy, the complimentary remarks of the clerks to each other, the frequent visitations of nurses and doctors, her life, as may be imagined, is anything but monotonous. Again, the preparation for committee meetings, often at very short notice, is quite exhilarating, and the sweet relief which is experienced when a hastily prepared meeting is at an end without anything dreadful having occurred is really inexpressible.

As compared with the commercial typist, the hospital typist has many advantages. Unless the hospital is a very ancient one, the office in which she works is usually clean and healthy, and hospital life is infinitely more interesting than commercial life. In comparison with hospital life, the routine in many business houses seems dry and monotonous. It is the human element in the hospital which makes it so interesting, and a sympathetic observer can learn more of human nature in twelve months of hospital life than in twelve years of ordinary commercial experience. In a hospital one gets to know life as it really is, and, though the knowledge is often fraught with heartache and sadness, for "he that increaseth knowledge increaseth sorrow," yet it is good to feel that even in a humble way one is helping in a great and noble work. There is a great deal of humour connected with hospital work, too, as anyone acquainted with hospital correspondence will readily admit. Letters are received from time to time which are replete with most refreshing humour; the spelling is unique, and the graphic diagnosis given by some ought to render a medical examination quite superfluous.

In every walk of life where a position of any merit is to be attained one must work long and strenuously, keep in touch with modern thought and progress, and even the humble typist is no exception to this rule.

(*Continued on p. 2, col. 3.*)

### Question for October.

### PATIENTS' WAITING-LISTS.

**Describe a simple method of recording names of patients awaiting admission, showing at a glance their proper turn for admission having regard both to period of waiting and the medical urgency of their respective cases.**

**A minimum payment of 10s. 6d. will be made for each published answer.)**

RULES.

The following rules must be observed:—

1. Contributions must be written on one side of the paper. Conciseness and terseness are desirable features. The MS. must bear the name and address of the sender and be accompanied by coupon to be cut from the back cover (inside page) of the current issue of THE HOSPITAL. A pseudonym must be chosen if the name is not to be published.

2. Contributions must be addressed to the Editor of THE HOSPITAL Institutional Worker Supplement, 28 & 29 Southampton Street, Strand, London, W.C. 2, should reach him before the end of the current month, and be marked in the left-hand corner "Facts and Figures."

### QUESTIONS INVITED.

IN connection with our Question Box, we offer every month five shillings each for the two best questions which are sent in for consideration. The questions may be on any institutional subject and concern any department, from the secretary's to the porter's, or the matron's to the domestic staff; the one essential is that they must be practical and deal with points that have a definite relation to hospital or institutional administration, upkeep, finance, or management. Points relating to artificers' work, housekeeping, the laundry, out-patients, and other practical matters will be welcome. It is hoped that thus, when difficult or doubtful points arise in the course of their work, institutional workers will be encouraged to put them in the form of a question and send them to the Editor, THE HOSPITAL Bureau of Information, 28 Southampton Street, Strand, W.C. 2, marked "Question Box."

The best questions will be published in due course and our readers will be given the opportunity of answering them. Thus all institutional workers may be able to co-operate with the view of helping the work and smoothing the difficulties of each other. In short, we want the Question Box of the *Institutional Worker* to be freely used by every worker habitually.

**The Editor will be glad to receive correspondence and to consider contributions upon all subjects relating to institutional work which affect the welfare of institutional workers.**

### ENQUIRIES AND ANSWERS.

### *THE SICK AND IN NEED.*

#### Convalescent Home on the South Coast.

WE fear from the brief particulars you give us that the patient you refer to is not suitable for a convalescent home. What was the cause of his paralysis. Provided that the nature of his illness does not exclude his admission, the Merchant Taylor's Convalescent Home at Bognor would probably be most suitable. You should write to the Clerk, Merchant Taylor's Company, 30 Threadneedle Street, London, E.C.—E. Q.

#### Home for Mentally Deficient.

WE think that in the circumstances your best course would be to apply to the Central Association for the Care of the Mentally Defective, Queen Anne's Chambers, Tothill Street, London, S.W. The Association would advise you as to the procedure necessary to secure the proper care of the boy.—H. F.

#### Home for Clergyman's Widow.

YOU might gain admission to one of the following homes, but it is quite possible that you will have to wait some time for a vacancy in any one of them: Somerset Hospital, Froxfield, Wiltshire; houses are appropriated to clergy widows in the following manner: Wilts, Berks, and Somerset, 10; London and Westminster, 5; Counties at large, 5. Widows eligible for nomination must be settled in some parish or district, whence they are selected, or whose last place of residence shall have been usually within such a district for twelve months next previous to vacancy. The hospital provides a home and a small annual allowance. Clergy Widow's Almshouses, Church Street, Ashbourne: this home is for widows of clergymen of the Established Church, without limit to diocese. The inmates receive a stipend of £45 a year. Bromley College, Bromley, Kent; a house and pension is provided for clergymen's widows whose age is not under fifty at time of election.—MRS. L.

### *EMPLOYMENT AND TRAINING.*

#### Dental X-ray Work.

POSSIBLY the Secretary of the School Dentists Society, c/o Messrs. John Bale, Sons, and Danielsson, Ltd., 88 Great Titchfield Street, W. 1, would be able to suggest some means whereby you might obtain further work in dental *x*-ray work. Write to him, enclosing stamped envelope for reply.—W. T. B.

#### Useful Handbook for Health Workers.

YOU will find "Residential Institutions in Connection with Maternity and Child Welfare in England and Wales" of value to you. Amongst other things it contains lists of homes for mothers and babies, maternity homes and hospitals, and convalescent homes for mothers and babies. It is published by the Health Ministry, and is obtainable from His Majesty's Stationery Office, Imperial House, Kingsway, London, W.C. 2. The price is 5d. post free.—HEALTH WORKER.

#### Mental Nursing.

THE course to pursue if you wish to take up mental nursing is to apply to the superintendent of the asylum or mental hospital you wish to enter. You will find a list of institutions in "How to Become a Nurse," published by The Scientific Press, Ltd., 28 and 29 Southampton Street, Strand, W.C. 2. This book gives much valuable information, and would repay reading. Vacancies are frequently advertised in the nursing and Local Government Board journals, as well as in the daily newspapers.—BETTY.

#### Training in Social Work.

THE full-time course on social training organised by the University of London begins in October of any year. The course occupies two sessions, and includes practical and theoretical work. Opportunities are also given for specialisation, such as welfare work. Write to the Secretary, London School of Economics, Clare Market, Portugal Street, Kingsway, London, W.C. 2, for a list of the course.—R. M. H.

#### Post-Graduate Course on Infant and Child Welfare.

A COURSE of twelve practical demonstrations on the management and Feeding of Infants and Young Children will be given by Dr. Eric Pritchard to qualified practitioners, commencing Tuesday, October 5, 1920, at the St. Marylebone General Dispensary. Students attending this course will have the opportunity of visiting the Nursery Training School, 1 Wellgarth Road, Golder's Green, on Saturday afternoons.—V. A.

### THE HOSPITAL TYPIST.

*(Continued from p. 1.)*

If she is to obtain a post of any interest and value, there is much to be done, and many lessons to be learned, which, alas! can only be gained by bitter experience. How well I remember my *début* into the hospital world—the nervous anxiety to please, the natural shrinking from observation, and how the great institution seemed a monstrous and overpowering whirl of intricate systems which I thought I should never understand; yet that same institution is to me now as fascinating and wonderful as it was then strange and dreadful. How well I remember with what a shaking hand I wrote my first letters. I am sure that no politician making his maiden speech, or *débutante* at a Drawing Room, suffered more than I did at that moment, and no victor rejoiced more than I at the successful completion of the day's work. I felt that I had successfully launched my small barquè on the sea of life.

